# Identifying Missed Opportunities for Routine Vaccination among People Who Use Drugs

**DOI:** 10.3390/ijerph18041447

**Published:** 2021-02-04

**Authors:** Paula M. Frew, Jay T. Schamel, Laura A. Randall, Adrian R. King, Ian W. Holloway, Katherine Burris, Anne C. Spaulding, HBOU Project Team

**Affiliations:** 1 UNLV School of Public Health, UNLV School of Medicine, and UNLV Population Health & Health Equity Initiative, University of Nevada, Las Vegas, NV 89154, USA; paula.frew@unlv.edu (P.M.F.); jayschamel@gmail.com (J.T.S.); laura.randall@unlv.edu (L.A.R.); Adrian.king@unlv.edu (A.R.K.); burrik2@unlv.nevada.edu (K.B.); 2 Department of Social Welfare, UCLA Luskin School of Public Affairs, Los Angeles, CA 90095, USA; holloway@luskin.ucla.edu; 3 Department of Epidemiology, Emory University, Atlanta, GA 30322, USA

**Keywords:** adult vaccination, vaccine missed opportunities, immunization, vaccine uptake, vaccine coverage, substance use, opioid crisis, social determinants, hidden populations, health equity

## Abstract

In the US, adult immunization coverage remains low, especially among vulnerable populations, as recent hepatitis A outbreaks have demonstrated. We studied the vaccination history variation among the US adults who use drugs by implementing a community-engaged research survey to identify reported immunization coverage, missed opportunities (MO), and places where immunizations might be delivered. Our analysis of a sample of 1127 participants recruited at community syringe exchanges in three cities identified higher overall vaccination receipt in Los Angeles compared to Atlanta or Las Vegas (e.g., HAV receipt 52.2% LA, 42.1% LV, 41.4% Atlanta). Overall, fewer participants reported having received HAV (45.9%), HBV (47.5%), or influenza (47.6%) vaccines than MMR (57.1%) or Td/Tdap (61.1%). Across sites, HAV receipt was higher for participants incarcerated ≥ 5 years (54.2% vs. 43.6% for those incarcerated < 5 years, 49.4% no incarceration history, *p* = 0.02). HBV receipt was higher among participants who were not intravenous drug users (56.1% vs. 46.0%, *p* = 0.03). Additionally, income >$20k predicted higher rates of MMR receipt (67.0% vs. 56.5%, *p* = 0.009), as did stable housing (62.8% vs. 54.3%, *p* = 0.01). To address the need to expand vaccine coverage among vulnerable adults, delivering vaccine at sites where persons who use drugs access services, or in correctional facilities, may be warranted.

## 1. Introduction

Recently released CDC surveillance has identified an ongoing problem among the US adults—the persistence of suboptimal vaccination despite public health efforts to achieve Healthy People 2020 immunization goals established to increase immunization rates and reduce preventable infectious diseases [1,2,3,4,5,6,7]. Notably, seasonal influenza vaccination remains very low at 45.3% coverage among adults aged 18 years and older during the 2018–2019 influenza season, well below the Healthy People 2020 goal of 70% for non-institutionalized adults [1,8,9,10]. Influenza vaccine coverage has decreased in recent years among whites and has declined overall among adults ≥ 65 years [4]. Other recommended vaccines for those over age 18 years including pneumococcal, Tdap, hepatitis A, and hepatitis B also fall below Healthy People goals [5]. In 2017, hepatitis A vaccination coverage was reported at 10.9% for adults over the age of 19 [11]. Hepatitis B coverage had a higher vaccination coverage among adults over 19 years of age reported at 25.8% [11]. 

In addition, adult vaccination differences have been observed based upon racial and ethnic identity, gender, age groups, and socioeconomic status [12]. Some studies have linked social determinants of health to adult vaccination disparities [13,14,15]. For example, factors such as health literacy, poverty/low socioeconomic status, residential area, and access to information, may be linked to vaccine-related (e.g., pneumococcal) decisions [13]. Among adults ages 19–49 years old, 16.3% of whites reported hepatitis A coverage compared to African Americans (11.7%) and Hispanics (13.4%) [11]. Hepatitis B vaccination coverage for adults ages 19–49 was reported at 27.3% for Hispanics, 30.7% for African Americans, and 36.6% for whites [11]. Others have observed that Medicare recipients, black, Hispanic, rural, and poorer beneficiaries are less likely to have obtained influenza vaccination compared to other groups [16]. 

In the US, over 10 million people aged 12 years or older misused opioids in 2018, [17] with most reporting additional prescription pain reliever misuse and 808,000 reporting additional heroin use [17]. Over the past 21 years, approximately 750,000 people have died as a result of a drug overdose [18]. In 2018 alone, 67,367 drug overdose fatalities occurred, with nearly 70% involving opioid use [19]. Despite these facts, the impact of the opioid epidemic in the US on public health far exceeds the morbidity and mortality statistics. Opioid misuse and opioid use disorders are associated with increased risk of community transmission of infectious diseases (i.e., *Staphylococcus aureus*, HIV, HCV) and vaccine-preventable infections (e.g., hepatitis A and influenza) [20,21,22]. 

Although overall adult vaccination coverage and uptake rates are well-known, as well as immunization rate correlates and risk factors, less information is available on specific segments of the adult population at increased risk for vaccine-preventable diseases, including people who use drugs (PWUD) in specific geographic areas [23,24], and those experiencing housing instability or homelessness [25,26,27]. We use the term “PWUD” referring to those who use illicit substances including opioids. While a few prior studies have examined Hepatitis A virus (HAV) and Hepatitis B virus (HBV) vaccination among PWUD in the US, these studies are often limited in geographic coverage and sample size [28,29,30]. Information on receipt of other recommended vaccinations among PWUD is limited [31]. With increased attention on the public health consequences associated with opioid use, and limited understanding of opioid users’ vaccination behaviors, this survey was launched to expand the thinking about how to address gaps in vaccine delivery within a harm reduction context [32,33]. Using a survey of PWUD recruited from needle exchange services in three US cities, we assessed self-reported vaccination coverage, vaccination patterns, and risk factors and correlates of non-vaccination among this vulnerable population. The findings will not only inform public health practice and policy but also will endeavor to mitigate the infectious disease risks and burdens faced by these populations at the intersection of multiple vulnerabilities.

## 2. Materials and Methods 

### 2.1. Survey Design and Sample

The data for this survey of vaccine receipt among PWUD are drawn from a larger survey designed to describe the characteristics and health behaviors of this population at multiple sites across the US. The project was reviewed and approved prior to protocol implementation by the UNLV IRB. Potential participants were recruited at sites providing syringe services in Atlanta, Georgia, Los Angeles, California, and Las Vegas, Nevada. The community partners chosen for this survey were well established in their communities (providing services for at least two years) and offered a variety of delivery modes, including mobile, mail, and brick-and-mortar services. 

Recruitment and data collection occurred from 20 May 2019 through 10 February 2020. Participants were recruited by trained community partner staff and were eligible to participate in the survey if they had accessed the community partner’s syringe services more than once, self-reported use of opioids at least once in prior six months, resided in targeted counties, were age 18–68, and were comfortable answering questions in either English or Spanish. Potential participants were excluded if they could not or did not provide informed consent, if they were found to be cognitively impaired at the time of survey screening, or if they were unable to complete the self-administered screener. Screening and consent were conducted by interview with a community partner staff member, after which eligible participants were provided a Wi-Fi-enabled iPad to complete an electronic Qualtrics-based survey. Participants received health promotion items (e.g., travel size lotion, sunscreen, lip balm, gloves, t-shirts) and a nominal well-being items (e.g., hand sanitizer, socks, umbrella) for their time. Of 1368 partner clients recruited to screen for participation, 1127 were eligible, provided informed consent, and completed the survey.

### 2.2. Measures

#### 2.2.1. Vaccine Receipt

Participants were asked to report their vaccination status for several vaccines recommended for PWUD. Self-reported vaccination status was captured for the Hepatitis A (HAV), Hepatitis B (HBV), Influenza, Measles, Mumps, Rubella (MMR), and Tetanus, Diphtheria, Pertussis (Td/Tdap) vaccines. For each vaccine, participants could respond “Yes, I have received this vaccination”, “No, I have not received this vaccination”, or “I am not sure if I have received this vaccination.” Because individuals who are unvaccinated (“no”) or unsure of their vaccination status (“not sure”) are recommended to seek vaccination counseling [34,35], for the purposes of this analysis we consider the self-reported vaccination proportion to be the proportion of “yes” responses out of all “yes”, “no”, and “not sure” responses, rather than simply comparing “yes” responses to “no” responses. We did not ask if participants were up-to-date on vaccines requiring periodic booster doses (e.g., Tdap) or revaccination (e.g., influenza). For these period vaccines, receipt in lifetime was used as a simple proxy for influenza vaccination history.

#### 2.2.2. Sociodemographic Items

The survey also assessed a breadth of sociodemographic and socioeconomic items. Factors used for this analysis included age, race/ethnicity, gender, sexual orientation, education, household income, primary income source, homelessness/housing insecurity, and medical insurance.

#### 2.2.3. High-Risk Groups

This analysis also explores self-reported vaccination status among a few high-risk subpopulations, including participants who had ever traded sex for goods, a place to stay, money or drugs/alcohol, those who had intravenously injected drugs in the past 6 months, and those with histories of incarceration. Intravenous (IV) injection drug use in the past 6 months was determined by aggregating individual items asking participants whether they had used 13 different drug categories in the past 6 months, and to mark the methods by which those drugs were used (IV injection, non-IV injection, orally, nasally, or smoking). Participants were considered to have intravenously injected drugs if they marked “IV injection” to at least one drug category, and to not have intravenously injected drugs if they had a valid response to all 13 drugs and did not mark “IV injection” on any of them. The drug categories assessed included heroin, methadone, opiates/analgesics, barbiturates, sedatives, crack cocaine, powder cocaine, prescription amphetamines, street amphetamines, cannabis, hallucinogens, inhalants, spice, and bath salts.

### 2.3. Statistical Analyses

The analytic strategy included computation of reported vaccine receipt among our sample of PWUD for each vaccination for the whole sample and for each recruitment location, and population-level differences between recruitment locations assessed using chi-square tests. Patterns of vaccine receipt were explored by calculating pairwise percent concordance and Cohen’s kappa agreement measures between pairs of vaccines. 

To examine the relationship between vaccine receipt and sociodemographic and risk factors among our survey population, we stratified vaccine receipt percentages and computed differences across strata assessed by chi-square tests. The degree to which location difference could be explained by differences in measured sociodemographic and risk factors was assessed using a multivariable logistic regression model. We used the method of multiple imputation to address missing data in the multivariable logistic regression model while utilizing all available responses [36,37]. The multiple imputation model involves creating a large number of datasets with missing items filled randomly using information from other present items, applying the desired analysis to each imputed dataset, and then pooling the results while properly accounting for the variance introduced by estimation of missing data [37]. This maximizes the information use by allowing completed items to be incorporated into a multivariable analysis even when that participant may be missing responses to other variables, which let us leverage our high item completion rate (93%) to offset the relatively low number of participants who had completed every item in the multivariable model (47%). To apply the multiple imputation method, relationships between variables were iteratively estimated using the fully conditional specification (FCS) algorithm and these models subsequently used to randomly fill missing items in 100 imputed datasets [38]. For reproducibility, a fixed seed generated from Random.org’s true random number service was used during the generation of imputed datasets [39]. Ten iterations of the algorithm were run between the chosen imputed datasets to ensure independence. The FCS imputation model included all vaccine, sociodemographic, and risk factors, as well as individual drug indicators for IV injection use. The multiple imputation pooling algorithm was then used to compute the final multivariable odds ratios and 95% confidence intervals.

A type 1 error rate of α = 0.05 was used as a threshold for sufficiency of evidence for differences and odds ratios, and confidence intervals were calculated at a 95% confidence level. All analyses were conducted with SPSS 25 (IBM Corp, 2017, Armonk, NY, USA).

## 3. Results

### 3.1. Sociodemographic Characteristics

Table 1 displays the sociodemographic and risk-factor characteristics of the 1127 survey participants, overall and stratified by recruitment location. Participants were drawn across all age groups, with 22% aged 18 to 30, 52% aged 31 to 50, and 25% aged 51 or older. A greater proportion of Los Angeles participants were 51 or older, 44% compared to 8% in Las Vegas and 20% in Atlanta. Overall, the survey captured a spectrum of racial/ethnic identities, with 20% identifying Hispanic (and not multiracial), 42% as non-Hispanic White alone, 21% as Non-Hispanic Black alone, and 12% as other identities or multiracial. A majority of participants identified as male (63% vs. 36% female), and 76% identified as straight/heterosexual vs. 22% as gay, lesbian, bisexual, or other. Forty-one percent of participants had attended at least some college or technical school. A large majority of participants (78%) reported household income of less than $20,000, 37% reported public benefits or disability as their primary source of income (and 20% marked “other”), and 60% reported that they were homeless or housing insecure. Only 4% of participants reported possession of private insurance; most (64%) had only public or other insurance (Medicaid/Medicare/Veterans) and 24% had no insurance. Lack of insurance was highest in Atlanta (65%). Thirty-one percent of respondents reported having sex for goods, a place to stay, money, or drugs/alcohol at some point during their lives, with the highest rate among Atlanta participants (45%). Most participants reported intravenous injection of drugs in the past 6 months (80%), with the highest rates among Las Vegas participants (68% vs. 57% in Los Angeles and 67% in Atlanta). Twenty percent of respondents had been incarcerated for 5 or more years in their lifetime, with 31% of Los Angeles participants reported 5+ years compared to 12% in Las Vegas and 15% in Atlanta. 

### 3.2. Vaccination Receipt

Within our sample of PWUD, reported vaccine receipt varied between vaccines and across recruitment sites (Table 2). Overall, fewer participants reported having received HAV (45.9%), HBV (47.5%), or influenza (47.6%) vaccines than MMR (57.1%) or Td/Tdap (61.1%). HAV uptake varied across recruitment location (Chi-square *p*-value = 0.005), with higher uptake among Los Angeles participants (52.2%) than those in Las Vegas (41.4%) or Atlanta (42.1%). HBV rates exhibited a similar pattern, with 53.4% vaccinated in Los Angeles compared to 44.4% in Las Vegas and 42.0% in Atlanta (Chi-square *p*-value = 0.02), as did influenza vaccination (52.1% in Los Angeles vs. 46.0% in Las Vegas and 42.1% in Atlanta; Chi-square *p*-value < 0.001). For both HAV and HBV, the differences between Los Angeles and Las Vegas and between Los Angeles and Atlanta are statistically significant (*p* < 0.05) while only the difference between Los Angeles and Atlanta is statistically significant for influenza vaccination. We did not see sufficient evidence for differing vaccination rates between recruitment locations for MMR (Chi-square *p*-value = 0.4) or Td/Tdap (Chi-square *p*-value = 0.2).

Reported vaccine receipt was highly correlated across all vaccines (Table 3), but some receipt patterns were reported more often than others. HAV and HBV receipt were most often reported together (92% concordance, Cohen’s kappa = 0.83), as were MMR and Td/Tdap (87% concordance, Cohen’s kappa 0.74). Other pairs of vaccines had concordance ranging from 72% (HAV/Tdap) to 76% (HAV/influenza) and Cohen’s kappa statistics from 0.46 (HAV/Tdap) to 0.51 (HAV/influenza).

### 3.3. Factors Associated with Vaccine Receipt

Table 4 reports vaccine receipt percentages (“yes” vs. “no” or “not sure”) stratified across sociodemographic and risk factors. Of these factors, education was most consistently associated with differences in vaccine receipt; participants who had attended at least some college or technical school reported higher rate of vaccination than those with high school diplomas/GEDs or those who did not finish high school/GED for HAV (50.8% vs. 42.1% and 43.5%, Chi-square *p*-value = 0.03), HBV (54.1% vs. 42.3% and 44.3%, *p* = 0.002), MMR (66.1% vs. 50.9% and 50.5%, *p* < 0.001), and Td/Tdap (69.1% vs. 55.7% and 55.3%, *p* < 0.001). HAV and HBV receipt also varied between participants with different primary income sources, with those primarily relying on public benefits or disability reporting 51.1% HAV uptake and 53.4% HBV uptake, compared to 46.2% HAV and 44.9% HBV uptake for those full, part-time, or self-employed, and 39.2% HAV and 43.2% HBV for those who marked “other” as their primary income source (HAV *p*-value = 0.018, HBV *p* = 0.023). Additionally, we have sufficient evidence suggesting HAV receipt was higher for participants incarcerated 5 or more years (54.2% vs. 43.6% for those incarcerated <5 years and 49.4% for those with no incarceration history, *p* = 0.02), and HBV receipt was higher among participants who did not inject drugs (56.1% vs. 46.0%, *p* = 0.03). MMR and Td/Tdap receipt differed by racial/ethnic identity (MMR *p*-value = 0.04, Td/Tdap *p*-value = 0.003), with non-Hispanic White alone participants reporting the highest rates of MMR (62.1%) and Td/Tdap (66.7%), compared to 55.3% and 58.4% among non-multiracial Hispanic participants, 53.4% and 52.2% for non-Hispanic Black alone participants, and 50.0%, 56.8% for those identifying as other or multiracial. Additionally, participants with income >$20k had higher rates of MMR receipt (67.0% vs. 56.5%, *p* = 0.009), as did those who were not currently homeless or housing insecure (62.8% vs. 54.3%, *p* = 0.01). We did not find evidence for differences in influenza vaccine receipt across strata in any of the observed sociodemographic or risk factors.

After adjusting for recruitment location and sociodemographic and risk factors in a multivariable model, education was the most consistent predictor of higher vaccine receipt within our sample population (Table 5). After adjustment, respondents with at least some college/technical experience were still more likely to report HAV (Odds Ratio 1.40 vs. high school/GED and OR 1.44 vs. less than high school), HBV (ORs 1.58 and 1.66), MMR (ORs 1.73 and 1.73), and Td/Tdap (ORs 1.73 and 1.82) vaccine receipt. Additionally, incarceration for 5 or more years compared to incarceration less than 5 years was predictive of HAV receipt in the multivariable model (OR 1.47). Race/ethnicity remains associated with MMR and Td/Tdap, with non-Hispanic Black alone participants reporting less receipt than non-Hispanic White alone for MMR (OR 0.67) and Td/Tdap (OR 0.59) and Other/Multiracial participants with reduced Td/Tdap rates (OR 0.59) vs. non-Hispanic White alone. In the multivariable model, participants with “other” listed as their primary income source were less likely to report Td/Tdap receipt than those relying on public benefits/disability. After adjustment for sociodemographic and risk factors, we still found evidence for a location effect in HAV vaccine receipt, with higher rates in Los Angeles than Las Vegas (OR 1.47), but we did not find sufficient evidence for other location differences after adjustment.

## 4. Discussion

Consistent with other reports and surveillance, this survey found that reported vaccination is lower than recommended Healthy People goals for people who use drugs (PWUD), a high-risk population with significant opportunity for coverage improvement via targeted access points [6,11]. We did observe that HAV and HBV vaccination levels reported by the participants are indeed higher than those for the general U.S. population (i.e., HBV: 25.8% for >3 doses and HAV: 10.9%) and similar to those for influenza [11]. However, as this population is at high risk for vaccine-preventable diseases such as viral hepatitis, the coverage gap remains a serious concern [20,21,22,40]. As expected, we found that HAV/HBV, influenza, and MMR/Tdap vaccination was associated with characteristics signifying its perceived acceptability (i.e., longstanding ACIP recommendation) [41]. We acknowledge that these observations might be due, in part, to participant recall bias, yet the findings likely reflect the degree of vaccine-specific acceptability and availability and highlights the need for targeted approaches to each type of vaccine in specific populations.

For this analysis, we examined if any differences existed between vaccination rates and related risk factors. The results do not suggest much variation in vaccine receipt based upon demographic, economic, or risk characteristics [42,43,44,45]. Irrespective of the risk status, there was significant room for improvement in recommended adult vaccination rates (overall HAV/HBV, influenza: 46–48%, MMR: 57%, Tdap: 61%). When we observed differences between groups, even the more-vaccinated groups were significantly under Healthy People recommended vaccination goals. Additionally, of note, reported influenza vaccination was relatively low across all groups suggesting that barriers with this population are more universal to racially and ethnically diverse adult populations facing housing and food insecurity as well as significant physical and mental comorbidities [46,47,48]. Yet, it is important to note that even for this population who face significant challenges in accessing healthcare services, the participants in our survey reported receiving at least one dose of HBV vaccination at double the rate of the general American population. For HAV, the rate reported among our survey group is 4.5 times higher than that observed in the US population. Amid this backdrop, and despite the challenges they face related to healthcare access, this at-risk population appears to have made much progress. Our findings reflect the need to meet this population “where they are at” to promote immunization. This points to places in community settings where they access services, supplies, and support offered at designated times and days when the population is present. These approaches will enable public health to reduce missed opportunities for future immunization. 

Overall, we observed geographic effects in relation to the uptake of specific vaccines. Participants in Los Angeles reported significantly higher HAV, HBV, and influenza vaccination than those in Las Vegas or Atlanta. We have previously reported on these types of effects with parents/guardians of pediatric populations wherein vaccine acceptance is linked to US census-designated regions [49]. This project also underscores the importance of monitoring geographic clustering effects and the need for intensive ground-level monitoring of immunization coverage to avert future outbreaks. It is especially important to identify the level of community coverage, with estimated herd immunity in these settings, as the findings reveal correlated vaccine receipt (i.e., HAV & HBV and MMR & Tdap). Thus, cross-promotion and coeducation about the importance of recommended adult vaccines has the potential not only to increase specific vaccinations (i.e., hepatitis A) needed to protect populations at the intersection of multiple vulnerabilities but also to increase the overall vaccine coverage [50,51,52]. Across the three cities, however, we found no significant differences for MMR or Tdap vaccination. As these vaccines are typically associated with childhood receipt or for infant cocooning purposes, it is not surprising that the adults in our sample recalled having received MMR or did not identify with having any pertussis risk given their adult circumstances [48,53,54,55].

With respect to sociodemographic vaccination correlates, this project found that higher educational attainment level corresponded with greater HAV, HBV, MMR, and Tdap vaccine receipt. Previous research has shown that educational attainment is associated with awareness of risk of vaccine preventable diseases, awareness of vaccination options, and with vaccine receipt itself [30,56]. Within this population at high risk for vaccine-preventable diseases, we believe the strength of this association reflects underlying health awareness and knowledge of the need for self-protective mechanisms while engaging in substance abuse [57,58]. Many programs condition syringe exchange opportunities, treatment, and recovery on receipt of health education about prevention of infectious diseases through accepted practices, guidelines, and program engagement [58,59]. Thus, it is highly likely that adults in this sample with college-level educational attainment are better prepared to understand and internalize these messages and adhere to vaccine recommendations than those with lower health literacy [60]. In treatment and recovery settings, as well as at syringe service programs, public health leaders should promote immunization education and provide standing or rotating clinical access to vaccines and be aware of the additional challenges in constructing materials suitable for all educational levels.

This survey identified differences in vaccine receipt among those of diverse racial and ethnic backgrounds. Specifically, the reported receipt of both MMR and Tdap vaccines in our models are higher for those who self-identified as non-Hispanic whites than those who identified as non-Hispanic black or multiracial/other cultural background. As issues of distrust in the medical system and in vaccines remains prevalent in the black/African American community [61,62,63], the reported rates are not unexpected nor are they inconsistent with general adult vaccination attitudes and behaviors [64]. Nonetheless, with the conflation of syndemic factors (e.g., substance use, homelessness/housing instability, and infectious disease burden) in frame for black/African American and multicultural members of the community, these findings underscore the urgent need to address the identified social determinants resulting in these suboptimal vaccination rates [65,66]. With diverse members of the community as immunization program leaders at places where clients congregate, seek services, and access support, greater opportunity exists to conduct effective vaccine dialogue and educational interventions that promote trust and confidence in immunization recommendations. 

Additionally, receipt of HAV and HBV vaccines strongly correlated with receipt of public benefits or disability support as a primary income source. As most of those in our sample who reported homelessness also received such benefits, past receipt of HAV vaccine may be linked to recent strong promotion of vaccination following the Advisory Committee on Immunization Practices (ACIP) recommendations for these communities [42,67] or to the increased exposure to public resources. This survey also identified that reported MMR receipt was higher for participants with >$20k household income and for those who are not currently homeless or housing insecure. This may be due to receipt of the vaccine in early childhood prior to the initiation of substance use in adulthood [61], or access to health services including immunization within community settings where measles has been a more widespread concern [58,61].

Additional location effects were observed with HAV vaccine receipt as the Los Angeles participants reporting greater levels of immunization against HAV compared to Las Vegas or Atlanta. The multivariate model accounting for location also found overall acceptance of HBV immunization to be higher for females than males. With robust vaccination campaigns in LA, and potentially greater points of access in the region, these effects have likely been realized from such concentrated public health efforts to reduce the burden of disease in areas we surveyed [57,68]. Correspondingly, we also identified those with significant (5+ years) incarceration history received HAV vaccine in greater numbers compared to those who had reduced sentences in facilities such as jails and prisons. In the multivariable models adjusting for these factors and recruitment location, we similarly found greater receipt of HAV vaccination among those with longer confinement. This finding indicates that jails, prisons, and detention centers may be missed opportunity locations to reach and deliver recommended adult vaccines [40,69]. 

Considering higher rates of vaccine-preventable diseases experienced by PWUD, and the gaps in their vaccination coverage, public policy interventions ideally would promote vaccination among PWUD. For example, state and local policies that prioritize harm reduction services for PWUD, including syringe service programs and safer consumption sites, may also provide guidance and resources for vaccination. Consideration of PWUD in the design of services and offering direct block grants to support those services could help close the vaccination coverage gap [42]. Additionally, ensuring that institutions that regularly serve PWUD (e.g., jails, syringe exchange programs) have access to resources to promote linking PWUD to vaccination services could improve vaccination coverage and reduce disease burden [70].

Limitations include the self-reported nature of data. We were unable to confirm vaccination status or timing with health department or state registries or medical records due to anonymity of participation and the expense and labor required for registry confirmation. However, we incorporated several measures to ensure that the self-reported data were as accurate as possible, including ensuring that participants were clear-minded during recruitment, implementing in-person screening and consent to ensure participants would be able to understand and respond to survey questions, and supervised, but private at-location survey administration. As the survey did not ask when participants received recommended adult vaccinations, there is no way to know what percentage of those happened earlier versus later in their life. Furthermore, we could not confirm whether vaccinations requiring periodic redosing (Tdap) were up-to-date, or patterns of seasonal influenza vaccination. For confidentiality reasons, this survey did not ask participants where they received the recommended adult vaccinations (e.g., HAV and HBV). Thus, we acknowledge the difficulty interpreting the reported high rates of immunization among those incarcerated for five or more years. Furthermore, the geographic location where the participants received their vaccinations (i.e., city) is unknown, making it difficult to interpret differences by location. Future research in this domain therefore should include information on these specifics. While this analysis assesses self-reported vaccination among PWUD who used syringe exchange services in three cities across the US, these results may not directly generalize to other areas or to clients of other syringe exchanges. However, these findings are important to consider in the context of mass vaccination against SARS-CoV-2, providing useful guidance to health authorities. 

## 5. Conclusions

In this project, we observed characteristically low overall estimated vaccination uptake (e.g., HAV/HBV and influenza: 46–48%, MMR: 57%, Tdap: 61%) among a highly vulnerable, geographically and racially/ethnically diverse population of PWUD recruited from syringe exchange services. We observed differences in reported immunization based upon educational attainment and adult age group suggesting that more targeted information is needed that accounts for health literacy and recommendations for specific age ranges. In addition, we found evidence of geographic variation as Los Angeles had significantly higher reported HAV, HBV, and influenza vaccination rates than Las Vegas or Atlanta. Access to recommended immunizations may be more readily available in Los Angeles, as it is characterized by more extensive healthcare access points and social and healthcare service utilization in its communities compared to other US cities. Currently, both Los Angeles and Las Vegas, unlike Atlanta, are Medicaid expansion states. Additionally, we identified those facing incarceration or at risk for recidivism may have access to vaccines in jails, prison, or detention that may not be available to others using substances in the community. Thus, this project offers insight on how to address adult immunization gaps by leveraging community resources to ensure that these “hidden populations” hear the message and are guided toward receipt of recommended adult vaccinations.

## Figures and Tables

**Table 1:** Sociodemographic characteristics of participants, by recruitment location.

Characteristic	Total	Las Vegas	Los Angeles	Atlanta
	*N = 1127*	*N = 414*	*N = 465*	*N = 248*
	*n* (%)	*n* (%)	*n* (%)	*n* (%)
**Age**				
18 to 30	252 (22.4%)	140 (33.8%)	49 (10.5%)	63 (25.4%)
31 to 50	590 (52.4%)	243 (58.7%)	212 (45.6%)	135 (54.4%)
51 or older	285 (25.3%)	31 (7.5%)	204 (43.9%)	50 (20.2%)
**Race/Ethnicity**				
Hispanic, not multiracial	226 (20.1%)	65 (15.7%)	141 (30.3%)	20 (8.1%)
Non-Hispanic White alone	478 (42.4%)	267 (64.5%)	107 (23.0%)	104 (41.9%)
Non-Hispanic Black alone	232 (20.6%)	20 (4.8%)	112 (24.1%)	100 (40.3%)
Other and/or multiracial	140 (12.4%)	51 (12.3%)	66 (14.2%)	23 (9.3%)
*Don’t know, decline to answer, or missing*	*51 (4.5%)*	*11 (2.7%)*	*39 (8.4%)*	*1 (0.4%)*
**Gender**				
Male	705 (62.6%)	252 (60.9%)	299 (64.3%)	154 (62.1%)
Female	406 (36.0%)	162 (39.1%)	157 (33.8%)	87 (35.1%)
Other	5 (0.4%)	0 (0.0%)	0 (0.0%)	5 (2.0%)
*Decline to answer or missing*	*11 (1.0%)*	*0 (0.0%)*	*9 (1.9%)*	*2 (0.8%)*
**Sexual Orientation**				
Straight	853 (75.7%)	321 (77.5%)	384 (82.6%)	148 (59.7%)
Gay/Lesbian/Bisexual/Other	248 (22.0%)	87 (21.0%)	65 (14.0%)	96 (38.7%)
*Decline to answer or missing*	*26 (2.3%)*	*6 (1.4%)*	*16 (3.4%)*	*4 (1.6%)*
**Education**				
Less than high school/GED	244 (21.7%)	67 (16.2%)	128 (27.5%)	49 (19.8%)
High school/GED	404 (35.8%)	159 (38.4%)	155 (33.3%)	90 (36.3%)
At least some college/technical	459 (40.7%)	185 (44.7%)	168 (36.1%)	106 (42.7%)
*Decline to answer or missing*	*20 (1.8%)*	*3 (0.7%)*	*14 (3.0%)*	*3 (1.2%)*
**Household Income**				
$20, 000 or less	770 (78.5%)	257 (71.2%)	356 (86.6%)	157 (75.1%)
Greater than $20, 000	211 (21.5%)	104 (28.8%)	55 (13.4%)	52 (24.9%)
**Primary Income Source**				
Employment (full/part/self)	342 (30.3%)	153 (37.0%)	93 (20.0%)	96 (38.7%)
Public benefits or disability	423 (37.5%)	85 (20.5%)	293 (63.0%)	45 (18.1%)
Other	223 (19.8%)	111 (26.8%)	47 (10.1%)	65 (26.2%)
*Decline to answer or missing*	*139 (12.3%)*	*65 (15.7%)*	*32 (6.9%)*	*42 (16.9%)*
**Currently Homeless or Housing Insecure**				
Yes	680 (60.3%)	199 (48.1%)	322 (69.2%)	159 (64.1%)
No	376 (33.4%)	195 (47.1%)	119 (25.6%)	62 (25.0%)
*Not sure, decline to answer, or missing*	*71 (6.3%)*	*20 (4.8%)*	*24 (5.2%)*	*27 (10.9%)*
**Medical Insurance**				
Private insurance	48 (4.3%)	21 (5.1%)	17 (3.7%)	10 (4.0%)
Medicaid/Medicare/Veteran’s/Other, but no private insurance	716 (63.5%)	306 (73.9%)	360 (77.4%)	50 (20.2%)
No insurance	270 (24.0%)	61 (14.7%)	47 (10.1%)	162 (65.3%)
*Decline to answer, not sure, inconsistent response, or missing*	*93 (8.3%)*	*26 (6.3%)*	*41 (8.8%)*	*26 (10.5%)*
**Ever traded sex for goods, a place to stay, money, or drugs/alcohol**				
Yes	331 (30.8%)	128 (31.5%)	95 (22.1%)	108 (45.2%)
No	670 (62.3%)	260 (64.0%)	294 (68.4%)	116 (48.5%)
*Not sure or missing*	*74 (6.9%)*	*18 (4.4%)*	*41 (9.5%)*	*15 (6.3%)*
**Intravenous injection drug use in past 6 months**				
Yes	765 (67.9%)	331 (80.0%)	267 (57.4%)	167 (67.3%)
No	150 (13.3%)	43 (10.4%)	75 (16.1%)	32 (12.9%)
*Inconsistent response or missing*	*212 (18.8%)*	*40 (9.7%)*	*123 (26.5%)*	*49 (19.8%)*
**Total time incarcerated**				
No incarceration history	164 (14.6%)	53 (12.8%)	75 (16.1%)	36 (14.5%)
Incarcerated, <5 years	613 (54.4%)	277 (66.9%)	186 (40.0%)	150 (60.5%)
Incarcerated 5+ years	231 (20.5%)	50 (12.1%)	145 (31.2%)	36 (14.5%)
*Missing*	*119 (10.6%)*	*34 (8.2%)*	*59 (12.7%)*	*26 (10.5%)*

Bolded titles with gray backgrounds indicate the sociodemographic characteristic subsequently stratified by level. Italicized rows indicate responses considered to be missing in subsequent analyses.

**Table 2:** Vaccine receipt by recruitment site.

Vaccine	Total	Las Vegas	Los Angeles	Atlanta	Chi-Square
	*N = 1127*	*N = 414*	*N = 465*	*N = 248*	
	*n* (%)	*n* (%)	*n* (%)	*n* (%)	*p*-value
**HAV** *(missing = 78)*					
yes	482 (45.9%)	163 (41.4%)	223 (52.2%)	96 (42.1%)	
no	323 (30.8%)	121 (30.7%)	125 (29.3%)	77 (33.8%)	0.005 *
not sure	244 (23.3%)	110 (27.9%)	79 (18.5%)	55 (24.1%)	
**HBV** *(missing = 106)*					
yes	485 (47.5%)	174 (44.4%)	219 (53.4%)	92 (42.0%)	
no	312 (30.6%)	119 (30.4%)	118 (28.8%)	75 (34.2%)	0.017 *
not sure	224 (21.9%)	99 (25.3%)	73 (17.8%)	52 (23.7%)	
**Influenza** *(missing = 114)*					
yes	482 (47.6%)	179 (46.0%)	210 (52.1%)	93 (42.1%)	
no	387 (38.2%)	134 (34.4%)	147 (36.5%)	106 (48.0%)	0.000 *
not sure	144 (14.2%)	76 (19.5%)	46 (11.4%)	22 (10.0%)	
**MMR** *(missing = 114)*					
yes	578 (57.1%)	217 (55.6%)	230 (57.1%)	131 (59.5%)	
no	227 (22.4%)	84 (21.5%)	89 (22.1%)	54 (24.5%)	0.370
not sure	208 (20.5%)	89 (22.8%)	84 (20.8%)	35 (15.9%)	
**Td/Tdap** *(missing = 123)*					
yes	613 (61.1%)	240 (62.2%)	244 (61.3%)	129 (58.6%)	
no	214 (21.3%)	70 (18.1%)	86 (21.6%)	58 (26.4%)	0.157
not sure	177 (17.6%)	76 (19.7%)	68 (17.1%)	33 (15.0%)	

* *p*-value < 0.05. Bolded titles with gray background indicate the vaccine for which the responses are presented in the subsequent rows.

**Table 3:** Patterns of vaccine receipt (*N* = 1127).

**Agreement Between Receipt of Vaccines (Cohen’s Kappa)**					
	**HAV**	**HBV**	**Influenza**	**MMR**	**Td/Tdap**
**HAV**	—	0.83	0.51	0.49	0.46
**HBV**	0.83	—	0.49	0.48	0.47
**Influenza**	0.51	0.49	—	0.49	0.50
**MMR**	0.49	0.48	0.49	—	0.74
**Tdap**	0.46	0.47	0.50	0.74	—
**Percent Agreement Between Receipt of Vaccines**					
	**HAV**	**HBV**	**Influenza**	**MMR**	**Td/Tdap**
**HAV**	—	92%	76%	74%	72%
**HBV**	92%	—	75%	74%	73%
**Influenza**	76%	75%	—	74%	75%
**MMR**	74%	74%	74%	—	87%
**Tdap**	72%	73%	75%	87%	—

**Table 4:** Percentages of reported vaccine receipt ^†^ by sociographic and risk factors (*N* = 1127) ^††.^

Characteristic	HAV	HBV	Influenza	MMR	Td/Tdap
	*n* (%)	*n* (%)	*n* (%)	*n* (%)	*n* (%)
**Age**					
18 to 30	101 (42.4%)	106 (45.1%)	108 (46.0%)	120 (51.3%)	139 (58.4%)
31 to 50	261 (46.8%)	261 (47.8%)	247 (46.4%)	309 (57.4%)	327 (61.8%)
51 or older	120 (47.4%)	118 (49.2%)	127 (51.6%)	149 (61.8%)	147 (62.0%)
*Chi-square test*	*p = 0.459*	*p = 0.661*	*p = 0.342*	*p = 0.065*	*p = 0.629*
**Race/Ethnicity**					
Hispanic, not multiracial	105 (49.3%)	104 (50.5%)	100 (49.0%)	114 (55.3%)	118 (58.4%)
Non-Hispanic White alone	205 (44.8%)	212 (46.6%)	200 (44.6%)	280 (62.1%)	303 (66.7%)
Non-Hispanic Black alone	87 (42.4%)	87 (45.5%)	101 (51.3%)	101 (53.4%)	96 (52.2%)
Other and/or multiracial	63 (47.0%)	62 (47.3%)	58 (46.0%)	64 (50.0%)	71 (56.8%)
*Chi-square test*	*p = 0.525*	*p = 0.762*	*p = 0.420*	p = 0.037 *	p = 0.003 *
**Gender**					
Male	288 (44.3%)	284 (45.2%)	296 (46.8%)	346 (55.5%)	371 (60.2%)
Female	188 (48.3%)	198 (51.8%)	181 (48.7%)	229 (59.9%)	237 (62.4%)
Other	4 (80.0%)	2 (40.0%)	2 (50.0%)	2 (66.7%)	2 (66.7%)
*Fisher’s exact test*	*p = 0.155*	*p = 0.123*	*p = 0.851*	*p = 0.353*	*p = 0.784*
**Sexual Orientiation**					
Straight	371 (46.7%)	368 (47.5%)	367 (47.5%)	435 (56.1%)	468 (60.9%)
Gay/Lesbian/Bisexual/Other	108 (45.2%)	113 (48.9%)	114 (50.0%)	139 (62.1%)	142 (64.0%)
*Chi-square test*	*p = 0.688*	*p = 0.702*	*p = 0.513*	*p = 0.114*	*p = 0.414*
**Education**					
Less than high school/GED	97 (43.5%)	94 (44.3%)	98 (47.1%)	103 (50.5%)	114 (55.3%)
High school/GED	160 (42.1%)	157 (42.3%)	163 (43.7%)	190 (50.9%)	204 (55.7%)
At least some college/technical	222 (50.8%)	232 (54.1%)	217 (51.3%)	283 (66.1%)	293 (69.1%)
*Chi-square test*	p = 0.031 *	p = 0.002 *	*p = 0.099*	p = 0.000 *	p = 0.000 *
**Household Income**					
$20, 000 or less	328 (45.2%)	327 (46.3%)	337 (47.3%)	401 (56.5%)	429 (60.9%)
Greater than $20, 000	98 (50.0%)	100 (51.8%)	97 (51.6%)	126 (67.0%)	122 (65.6%)
*Chi-square test*	*p = 0.230*	*p = 0.170*	*p = 0.298*	p = 0.009 *	*p = 0.245*
**Primary Income Source**					
Employment (full/part/self)	151 (46.2%)	140 (44.9%)	148 (47.7%)	184 (59.0%)	196 (63.2%)
Public benefits or disability	201 (51.1%)	205 (53.4%)	200 (52.4%)	227 (59.7%)	242 (63.9%)
Other	83 (39.2%)	89 (43.2%)	86 (42.6%)	106 (52.2%)	108 (54.3%)
*Chi-square test*	p = 0.018 *	p = 0.023 *	*p = 0.075*	*p = 0.187*	*p = 0.059*
**Currently Homeless or Housing Insecure**					
Yes	297 (46.3%)	290 (46.8%)	289 (46.9%)	333 (54.3%)	359 (59.1%)
No	161 (45.4%)	171 (49.0%)	169 (48.6%)	221 (62.8%)	229 (65.4%)
*Chi-square test*	*p = 0.783*	*p = 0.506*	*p = 0.623*	p = 0.011 *	*p = 0.054*
**Medical Insurance**					
Private insurance	26 (55.3%)	28 (60.9%)	25 (53.2%)	31 (67.4%)	32 (69.6%)
Medicaid/Medicare/Veteran’s/Other, but no private insurance	317 (47.5%)	320 (48.9%)	322 (49.5%)	378 (58.3%)	395 (61.7%)
No insurance	101 (39.5%)	103 (41.5%)	101 (41.2%)	133 (54.1%)	151 (61.1%)
*Chi-square test*	p = 0.037 *	p = 0.025 *	*p = 0.062*	*p = 0.201*	*p = 0.544*
**Ever traded sex for goods, a place to stay, money, or drugs/alcohol**					
Yes	145 (46.3%)	157 (51.5%)	147 (49.7%)	184 (61.5%)	189 (63.6%)
No	290 (45.9%)	286 (46.1%)	291 (47.0%)	337 (54.7%)	369 (60.5%)
*Chi-square test*	*p = 0.898*	*p = 0.126*	*p = 0.453*	*p = 0.050*	*p = 0.361*
**Intravenous injection drug use in past 6 months**					
Yes	325 (44.6%)	330 (46.0%)	334 (46.8%)	424 (59.3%)	456 (64.2%)
No	74 (52.5%)	78 (56.1%)	76 (54.7%)	81 (58.3%)	77 (57.0%)
*Chi-square test*	*p = 0.085*	p = 0.028 *	*p = 0.091*	*p = 0.822*	*p = 0.113*
**Total time incarcerated**					
No incarceration history	76 (49.4%)	73 (48.7%)	74 (49.7%)	85 (57.8%)	86 (57.7%)
Incarcerated, <5 years	257 (43.6%)	268 (46.9%)	266 (46.9%)	334 (58.5%)	356 (63.2%)
Incarcerated 5+ years	116 (54.2%)	116 (54.5%)	112 (52.3%)	126 (59.7%)	137 (66.5%)
*Chi-square test*	p = 0.022 *	*p = 0.166*	*p = 0.385*	*p = 0.930*	*p = 0.237*

^†^ Vaccine receipt percentage is “yes” responses out of all “yes”, “no”, or “not sure” responses. ^††^ The number of participants in each strata for each variable are available in Table 1. * *p*-value < 0.05. Rows containing Chi-square tests for differences in vaccine receipt between different levels of the indicated characteristic are labeled and italicized. Bolded titles with gray backgrounds indicate the sociodemographic characteristic subsequently stratified by level.

**Table 5:** Multivariable logistic associations between vaccine receipt ^†^ and sociodemographic and risk factors (*N* = 1127).

	Characteristic	HAV	HBV	Influenza	MMR	Td/Tdap
		OR (95% CI)	OR (95% CI)	OR (95% CI)	OR (95% CI)	OR (95% CI)
**Location**						
	Los Angeles vs. Las Vegas	1.47 * (1.04, 2.09)	1.39 (0.98, 1.98)	1.14 (0.80, 1.61)	1.20 (0.84, 1.72)	1.10 (0.76, 1.59)
	Atlanta vs. Las Vegas	1.24 (0.81, 1.89)	0.95 (0.62, 1.45)	0.74 (0.48, 1.13)	1.44 (0.92, 2.25)	0.87 (0.56, 1.36)
	Atlanta vs. Los Angeles	0.84 (0.55, 1.29)	0.68 (0.44, 1.05)	0.65 (0.42, 1.00)	1.20 (0.76, 1.89)	0.79 (0.51, 1.24)
**Age**						
	31 to 50 vs. 18 to 30	1.07 (0.77, 1.48)	0.99 (0.72, 1.37)	0.94 (0.68, 1.31)	1.19 (0.85, 1.65)	1.07 (0.77, 1.50)
	51 or older vs. 18 to 30	0.92 (0.60, 1.41)	0.89 (0.58, 1.39)	1.03 (0.67, 1.59)	1.55 (1.00, 2.41)	1.13 (0.71, 1.79)
	51 or older vs. 31 to 50	0.86 (0.61, 1.22)	0.90 (0.63, 1.29)	1.09 (0.77, 1.55)	1.31 (0.91, 1.88)	1.05 (0.72, 1.54)
**Race/Ethnicity**						
	Non-Hispanic White alone vs. Hispanic, not multiracial	0.92 (0.64, 1.31)	0.93 (0.65, 1.34)	0.87 (0.61, 1.24)	1.27 (0.88, 1.83)	1.40 (0.96, 2.04)
	Non-Hispanic Black alone vs. Hispanic, not multiracial	0.73 (0.48, 1.10)	0.90 (0.59, 1.37)	1.16 (0.76, 1.76)	0.85 (0.55, 1.31)	0.82 (0.53, 1.27)
	Other and/or multiracial vs. Hispanic, not multiracial	0.84 (0.54, 1.33)	0.85 (0.54, 1.35)	0.83 (0.52, 1.32)	0.72 (0.45, 1.15)	0.94 (0.59, 1.50)
	Non-Hispanic Black alone vs. Non-Hispanic White alone	0.79 (0.54, 1.16)	0.97 (0.66, 1.42)	1.33 (0.91, 1.95)	0.67 * (0.45, 0.99)	0.59 ** (0.40, 0.87)
	Other and/or multiracial vs. Non-Hispanic White alone	0.92 (0.61, 1.39)	0.91 (0.60, 1.38)	0.96 (0.63, 1.45)	0.57 ** (0.37, 0.86)	0.67 (0.44, 1.02)
	Other and/or multiracial vs. Non-Hispanic Black alone	1.16 (0.72, 1.86)	0.94 (0.59, 1.51)	0.72 (0.45, 1.16)	0.85 (0.53, 1.37)	1.14 (0.70, 1.86)
**Gender ^††^**						
	Female vs. Male	1.27 (0.96, 1.68)	1.33 * (1.00, 1.77)	1.10 (0.83, 1.46)	1.11 (0.83, 1.48)	1.04 (0.77, 1.40)
**Sexual Orientiation**						
	Gay/Lesbian/Bisexual/Other vs. Straight	0.87 (0.62, 1.22)	0.94 (0.67, 1.32)	1.12 (0.80, 1.57)	1.11 (0.78, 1.57)	1.08 (0.75, 1.54)
**Education**						
	High school/GED vs. Less than high school/GED	1.03 (0.73, 1.45)	1.05 (0.74, 1.49)	0.94 (0.66, 1.33)	1.00 (0.70, 1.42)	1.05 (0.74, 1.50)
	At least some college/technical vs. Less than high school/GED	1.44 * (1.02, 2.04)	1.66 ** (1.17, 2.36)	1.25 (0.89, 1.76)	1.73 ** (1.21, 2.48)	1.82 ** (1.27, 2.61)
	At least some college/technical vs. High school/GED	1.40 * (1.05, 1.88)	1.58 ** (1.19, 2.11)	1.33 (1.00, 1.78)	1.73 *** (1.29, 2.33)	1.73 *** (1.27, 2.35)
**Household Income**						
	Greater than $20, 000 vs. $20, 000 or less	1.20 (0.83, 1.71)	1.24 (0.86, 1.78)	1.25 (0.86, 1.80)	1.38 (0.94, 2.02)	1.11 (0.76, 1.64)
**Primary Income Source**						
	Public benefits or disability vs. Employment (full/part/self)	1.05 (0.74, 1.48)	1.27 (0.89, 1.82)	1.03 (0.72, 1.47)	1.07 (0.74, 1.54)	1.15 (0.79, 1.67)
	Other vs. Employment (full/part/self)	0.81 (0.56, 1.16)	0.96 (0.67, 1.39)	0.82 (0.57, 1.19)	0.82 (0.56, 1.19)	0.71 (0.48, 1.03)
	Other vs. Public benefits or disability	0.77 (0.52, 1.14)	0.76 (0.51, 1.12)	0.80 (0.54, 1.18)	0.77 (0.51, 1.15)	0.61 * (0.41, 0.92)
**Currently Homeless or Housing Insecure**						
	Yes vs. No	1.11 (0.83, 1.49)	0.97 (0.72, 1.30)	1.02 (0.76, 1.37)	0.84 (0.62, 1.14)	0.90 (0.66, 1.22)
**Medical Insurance**						
	Public, but no private vs. Private insurance	0.73 (0.39, 1.38)	0.64 (0.33, 1.21)	0.90 (0.48, 1.69)	0.93 (0.48, 1.83)	0.85 (0.43, 1.66)
	No insurance vs. Private insurance	0.58 (0.29, 1.14)	0.59 (0.29, 1.17)	0.84 (0.43, 1.65)	0.72 (0.35, 1.49)	0.99 (0.48, 2.05)
	No insurance vs. Public, but no private	0.79 (0.55, 1.13)	0.92 (0.64, 1.34)	0.93 (0.64, 1.34)	0.78 (0.53, 1.14)	1.17 (0.80, 1.72)
**Ever traded sex for goods, a place to stay, money, or drugs/alcohol**						
	Yes vs. No	1.11 (0.80, 1.53)	1.21 (0.88, 1.66)	1.15 (0.84, 1.57)	1.25 (0.90, 1.74)	1.09 (0.78, 1.52)
**Intravenous injection drug use in past 6 months**						
	Yes vs. No	0.83 (0.57, 1.22)	0.76 (0.51, 1.12)	0.89 (0.59, 1.32)	1.13 (0.72, 1.75)	1.24 (0.82, 1.89)
**Total time incarcerated**						
	Incarcerated, <5 years vs. No incarceration history	0.92 (0.63, 1.34)	1.04 (0.71, 1.53)	0.99 (0.67, 1.45)	1.03 (0.69, 1.53)	1.22 (0.82, 1.82)
	Incarcerated, 5+ years vs. No incarceration history	1.36 (0.86, 2.13)	1.37 (0.87, 2.17)	1.12 (0.71, 1.77)	1.05 (0.66, 1.67)	1.45 (0.91, 2.33)
	Incarcerated, 5+ years vs. Incarcerated, <5 years	1.47 * (1.03, 2.10)	1.32 (0.92, 1.88)	1.14 (0.79, 1.63)	1.03 (0.71, 1.47)	1.19 (0.81, 1.74)

^†^ Vaccine receipt percentage is “yes” responses out of all “yes”, “no”, or “not sure” responses. ^††^ Odds ratios not shown for Gender responses of “Other” because sample size is too small for meaningful interpretation (*n* = 10). * *p*-value < 0.05, ** *p*-value < 0.01, *** *p*-value < 0.001. Bolded titles with gray backgrounds indicate the sociodemographic characteristic subsequently stratified by level.

## Data Availability

Data are not publicly available due to privacy and confidentiality requirements.
